# Continuous Hemodynamic Monitoring in an Intact Rat Model of Simulated Diving

**DOI:** 10.3389/fphys.2019.01597

**Published:** 2020-01-13

**Authors:** Svein E. Gaustad, Timofei V. Kondratiev, Ingrid Eftedal, Torkjel Tveita

**Affiliations:** ^1^Møreforskning AS, Ålesund, Norway; ^2^Cardiovascular Research Group, Department of Medical Biology, UiT, The Arctic University of Norway, Tromsø, Norway; ^3^Department of Circulation and Medical Imaging, Norwegian University of Science and Technology, Trondheim, Norway; ^4^Anesthesia and Critical Care Research Group, Department of Clinical Medicine, UiT, The Arctic University of Norway, Tromsø, Norway; ^5^Division of Surgical Medicine and Intensive Care, University Hospital of North Norway, Tromsø, Norway

**Keywords:** cardiac function, decompression, diving, hyperbaric, left ventricular, rattus norvegicus

## Abstract

Cardiovascular risk is elevated in divers, but detailed information of cardiac function during diving is missing. The aim of this study was to apply an intact rat model with continuous monitoring of cardiac left ventricular (LV) function in a simulated diving experiment. Thirteen rats were inserted with a LV pressure–volume catheter and a pressure transducer in the femoral artery to measure hemodynamic variables, and randomly assigned to diving (*n* = 9) and control (*n* = 4) groups. The diving group was compressed to 600 kPa in air, maintained at pressure for 45 min (bottom phase), and decompressed to surface at 50 kPa/min. Data was collected before, during, and up to 60 min after exposure in the diving group, and at similar times in non-diving controls. During the bottom phase, stroke volume (SV) (−29%) and cardiac output (−30%) decreased, whereas LV end-systolic volume (+13%), mean arterial pressure (MAP) (+29%), and total peripheral resistance (TPR) (+72%) increased. There were no changes in LV contractility, stroke work, or diastolic function. All hemodynamic variables returned to baseline values within 60 min after diving. In conclusion, our simulated dive experiment to 600 kPa increased MAP and TPR to levels which caused a substantial reduction in SV and LV volume output. The increase in cardiac afterload demonstrated to take place during a dive is well tolerated by the healthy heart in our model, whereas in a failing heart this abrupt change in afterload may lead to acute cardiac decompensation.

## Introduction

In diving, the body must adjust to hyperbaric environments. There are changes in ambient pressure and breathing gas density, partial gas pressures and thermal conductivity, and added strain from physical exertion, psychological stress, and immersion (Mack and Lin, 1986; Lango et al., 1996; Leffler, 2001; Bennett and Rostain, 2003; Boussuges et al., 2007). As a consequence cardiovascular function is altered and the diver is exposed to; bradycardia, altered stroke volume (SV), reduced cardiac output (CO) and increased vascular resistance (Bove et al., 1974; Wilson et al., 1977; Shida and Lin, 1981; Molenat et al., 2004; Boussuges et al., 2006; Dujic et al., 2006). Human experiments have reported increased afterload, decrease in LV preload, and decrease in systolic performance after diving (Molenat et al., 2004; Boussuges et al., 2006). Animal experiments have documented increased cardiac contractility when assessed as maximal velocities of LV pressure rise (Stuhr et al., 1989; Risberg et al., 1995).

The sum of environmental stress factors encountered in diving can augment cardiovascular risk (Bove, 2011). While the vast majority of divers have no history of heart disease and active divers score better than the general population on known risk factors (Buzzacott et al., 2018), cardiovascular problems were reported as being prominent – second only to drowning – as cause of diver fatalities in the Divers Alert Network’s annual diving report (Buzzacott and Denoble, 2018). Prior human experimental studies of dive-induced cardiovascular changes have largely been limited to recording of baseline and post-dive data (Marabotti et al., 1999; Boussuges et al., 2006; Dujic et al., 2006). Thus, limited knowledge of functional parameters exist to describe the progression of cardiovascular changes taking place during the dive, which may be important for the tolerance of diving stress and outcome.

In this study, we report the set-up and application of a method for continuous monitoring of hemodynamic parameters in an intact, spontaneously breathing rat model. Simulated diving tests were done in an air-filled hyperbaric chamber for animal research. In this model we monitored LV pressure–volume relationship during all phases of a 600-kPa dry air dive: pre-dive, bottom phase, decompression, and post-dive.

## Materials and Methods

### Ethics

The research protocol was approved in advance by the Norwegian Council for Animal Research (approval ID 2111). All experimental procedures conformed to The European Convention for the Protection of Vertebrate Animals used for Experimental and Other Scientific Purposes (ETS 123).

### Animals

Adult female Sprague Dawley rats (Weight 259 ± 6.3 g) were obtained from Charles River Laboratories (Charles River Laboratories Inc., Sulzfeld, Germany). The animals were controlled at a 12 h dark-12 h light cycle with access to a standard rodent diet and water *ad libitum*. In order to limit stress, the same person handled the animals throughout the study and all experimental procedures were performed during the animals’ dark cycle.

### Anesthesia

Anesthesia was induced by an i.p. bolus injection of sodium pentobarbital (50 mg/kg + Fentanyl 0.05 mg/kg body weight). One hour later, a second bolus injection (25 mg/kg + Fentanyl 0.025 mg/kg body weight i.p.) was given. The rats remained under anesthesia for the duration of the invasive procedures and the experimental diving protocol, after which they were sacrificed by decapitation.

### Respiratory Support

To secure patent airways, the trachea was opened and a small-size metal tube (13G) inserted. Following surgery, the animals rested for 60 min to regain hemodynamic stability before the simulated diving commenced. All animals maintained spontaneous ventilation throughout the experiment.

### Catheterization for Hemodynamic Monitoring

A microtip pressure–volume (P–V) catheter (SPR-838, 2.0 F, Millar Instruments, Houston, TX, United States) was inserted into the right carotid artery and gently advanced into the LV under pressure guidance. A similar microtip catheter measuring the mean arterial pressure (MAP) was positioned in the left femoral artery. MAP, and pressure–volume signals were digitized at 1 kHz and recorded using ADInstruments LabChart DAQ software (AD Instruments, Hastings, United Kingdom). This software displays P–V raw data in a scrolling strip chart format and plots parameters against each other in real-time, ensuring continuous monitoring of P–V loop data. Data is saved to a hard disk on request. The recorded data; HR, maximal LV systolic pressure, LV end-diastolic pressure, maximal slope of LV systolic pressure increment (dP/dt_max_) and diastolic pressure decrement (dP/dt_min_), time constant of LV pressure decay, Tau (τ), SV, end-diastolic volume, end-systolic volume, CO, and stroke work were analyzed off-line using a cardiac pressure–volume analysis program (PVAN 3.6, Millar Instruments, Houston, TX, United States). Data were collected during steady-state baseline conditions, and during transient inferior vena cava occlusions performed to vary LV preload for determination of load-independent indices of systolic function such as preload recruitable stroke work (PRSW). Respiration frequency was determined by analyzing respiration-dependent, cyclic changes in the LV pressure curve.

### Temperature Monitoring

The animals were placed in a supine position on an electric heat pad to maintain appropriate temperature (37°C) throughout the experiment. Their core temperature was continuously monitored using a thermocouple wire with the sensor tip positioned in the lower 1/3 of the esophagus and connected to a digital thermometer (Thermoalert, Columbus Instruments, Columbus, OH, United States). For diving animals, the thermocouple controller was placed inside the hyperbaric chamber and monitored through an armored window.

### Simulated Diving Protocol

**FIGURE 1:**
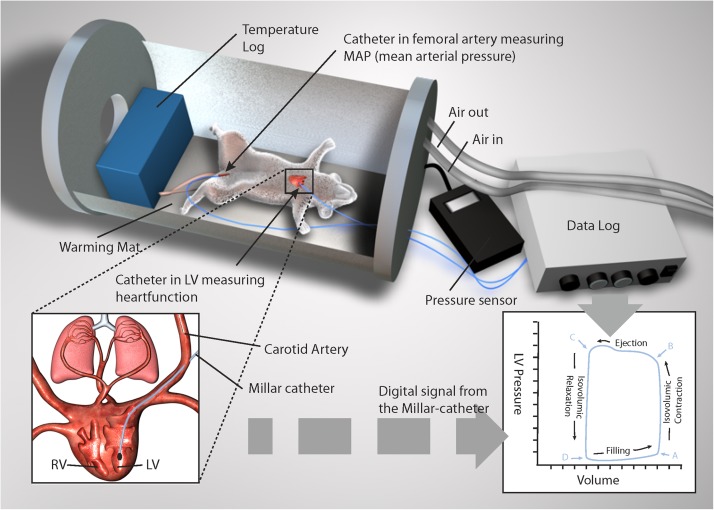
Depiction of pressure volume loop (PV-loop) recordings in an anesthetized rat during simulated diving. A Millar catheter was inserted into the left ventricle and femoral artery. Illustration made on request by MGS studios.

On each day of simulated diving, individual rats were randomly assigned to one of two groups: one diving (*n* = 9) and one non-diving control group (*n* = 4). Anesthetized rats were exposed to simulated diving one at a time in hyperbaric air in a pressure chamber for animal research (Sira Engineering, Trondheim, Norway). The experimental set-up is illustrated in Figure 1. Compression was done at a rate of 200 kPa min^–1^ to a pressure of 600 kPa (corresponding to 50 m water depth). The rat was maintained at pressure for 45 min while breathing air, before being decompressed to the surface over 10 min at a linear rate of 50 kPa min^–1^. The decompression was followed by a 60 min observation phase at surface pressure.

### Hemodynamic Data Recording

In the diving group, hemodynamic data were recorded at baseline (pre-dive), at 1, 2, 2.5, 5 min during the bottom phase, and subsequently every 10 min until 45 min when the bottom phase ended. During the decompression, recordings were done every 2 min, and during the post-dive observation phase, the rats were monitored after 2 min and then every 10 min up to 60 min. In the control group, hemodynamic parameters were recorded 60 (baseline), 120 and 180 min after the trachea surgery and hemodynamic catheterization.

### Vascular Bubble Detection

Immediately after the completion of decompression, the pulmonary artery and aorta of diving rats were insonated using a GE Vingmed Vivid i ultrasonic scanner (GE Vingmed Ultrasound, Horten, Norway), with a 10 MHz transducer as previously described (Wisloff and Brubakk, 2001). Ultrasound images were graded according to a method described previously (Eftedal and Brubakk, 1997). The insonation was repeated at 10-min intervals up 60 min post-dive.

### Statistics

Statistical analysis was done in SigmaPlot software (Systat Software Inc., San Jose, CA, United States). Normal distribution was checked using Shapiro–Wilk test. Within groups, hemodynamic data were analyzed by one-way repeated measures ANOVA for normal distributed variables and by Friedman repeated measures ANOVA on ranks for non-normal distributed variables. If the intragroup difference among values was greater than would be expected by chance, Dunnett’s test was used to evaluate differences between baseline value and responses to simulated diving at different time points. Differences were considered significant at *p* < 0.05. Results are presented as means ± SEM.

## Results

At baseline, after surgery and 60 min rest, there were no differences in hemodynamic variables between diving and non-diving animals. The stability of the model was demonstrated as no change in hemodynamic variables in time-matched, non-diving controls. All animals survived the experimental protocol.

### General Stress Assessment

**FIGURE 2:**
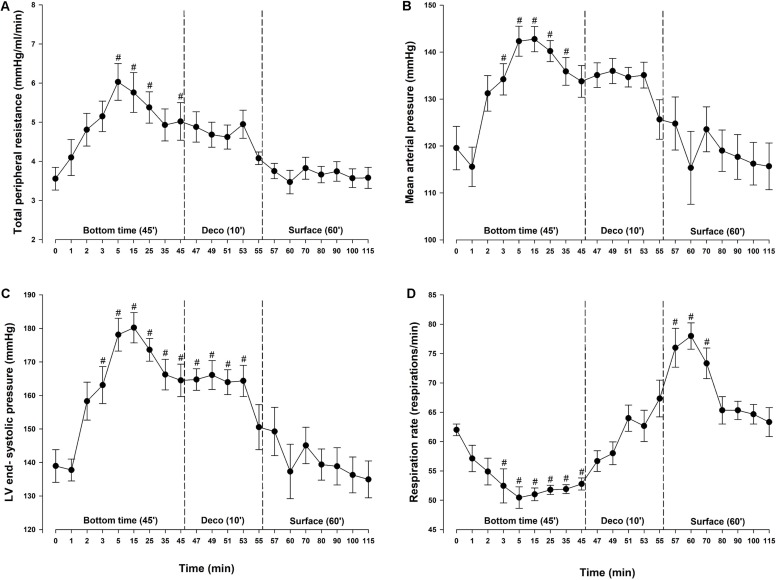
Determinants of cardiac afterload and respiration. **(A)** Total peripheral resistance (TPR), **(B)** mean arterial pressure (MAP), **(C)** left ventricular (LV) end-systolic pressure and **(D)** respiration rate (RR) in rats exposed to simulated diving (*n* = 9). Data were assessed using one-way repeated measures ANOVA for normal distributed variables and Friedman repeated measures ANOVA on ranks for non-normal distributed variables. Values are mean ± SEM. #*p* < 0.05 compared to intragroup baseline.

For diving animals, respiration rate (RR) was significantly decreased 2.5 min after reaching the bottom pressure at 600 kPa and remained below baseline throughout the bottom phase (Figure 2D). At surface, after decompression, RR exceeded baseline values before returning to normal within 15 min. Vascular bubbles were observed in two rats during the post-dive observation period; both with max bubble grade 2 on the Eftedal-Brubakk scale (Eftedal and Brubakk, 1997).

### LV Hemodynamic Function

**FIGURE 3:**
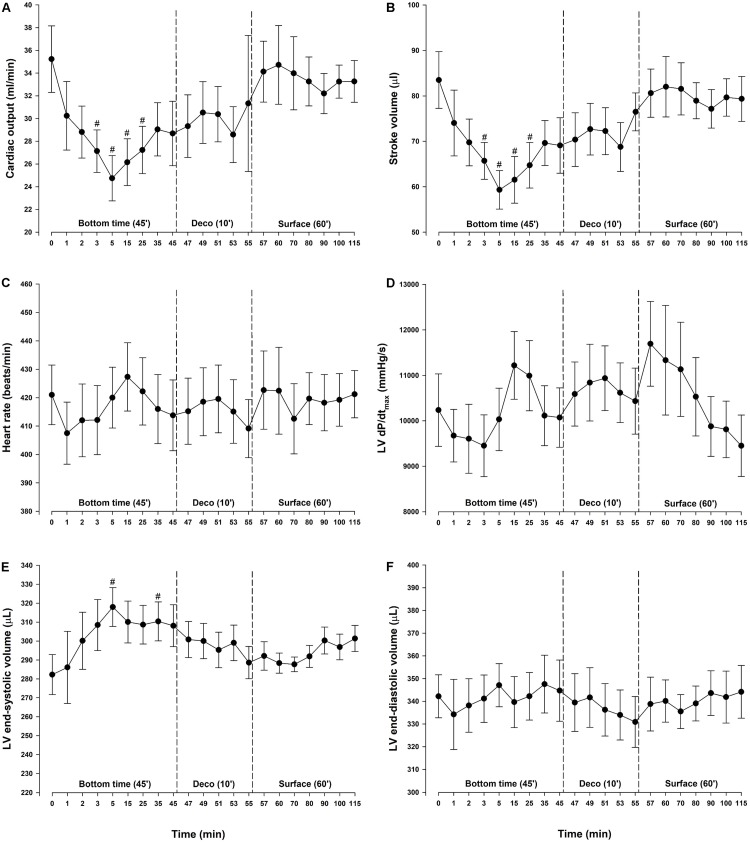
Cardiac output and its determinants. **(A)** Cardiac output (CO), **(B)** stroke volume (SV), **(C)** heart rate (HR), **(D)** maximal slope of left ventricular systolic pressure increment (LV dP/dt_max_), **(E)** left ventricular (LV) end-systolic volume and **(F)** left ventricular (LV) end-diastolic volume in rats exposed to simulated diving (*n* = 9). Data were assessed using one-way repeated measures ANOVA for normal distributed variables and Friedman repeated measures ANOVA on ranks for non-normal distributed variables. Values are mean ± SEM. #*p* < 0.05 compared to intragroup baseline.

During diving, MAP and total peripheral resistance (TPR) increased by 29 and 72%, respectively, and remained elevated throughout the bottom phase (Figures 2A,B). During the bottom phase a linear reduction in SV took place in parallel with a reduction in CO. At 5 min bottom time SV was decreased by 29% (Figure 3B). Heart rate remained stable throughout the experiment (Figure 3C) and thus the concurrent 30% reduction in CO (Figure 3A) was due to the decrease in SV only. During compression, following the abrupt increase in MAP and TPR, LV end-systolic volume increased to levels reaching significance at 5 (+13%) and 35 (+10%) -min bottom time, but remained unaltered during decompression (Figure 3E). A simultaneous elevation of LV end-systolic pressure (+29%) was measured during the bottom phase (Figure 2C). It remained elevated during decompression, before returning to normal at the surface. LV end-diastolic volume was unchanged throughout the simulated dive (Figure 3F). Stroke work (mmHg^.^μL), LV dP/dt_min_ (mmHg/s) and LV end-diastolic pressure (mmHg) were unchanged (data not shown). No change in cardiac contractility was detected when determined by calculating PRSW before compression (76.2 ± 5.9) and after decompression (68.5 ± 9.7) (data not shown). Another determinant of cardiac contractility, dP/dt_max_, was continuously measured but showed no change (Figure 3D).

## Discussion

The present experiment demonstrated an abrupt increase in LV cardiac afterload occuring already in the first 5 min of a 600-kPa simulated air dive, to an extent which reduced both SV and CO by ∼30%. The increase in afterload was substantiated as increases in MAP and TPR which caused a physiologic increase in LV end-systolic pressure and volume and a subsequent reduction in SV. The LV volume output remained reduced during the bottom phase but was reversed within 60 min post-dive. To the best of our knowledge, this is the first report of real-time hemodynamic monitoring in response to changes in ambient pressure.

Most studies of dive-induced cardiovascular changes have recorded hemodynamic variables at baseline and after the dive only. Some have reported cardiovascular responses to increased ambient pressures at discreet time points during the bottom phase, but not measured LV function continuously (Stuhr et al., 1989; Risberg et al., 1995; Molenat et al., 2004; Boussuges et al., 2007). In man, hyperbaric hyperoxic exposure up to 300 kPa air pressure produced no change in CO after 15 min, but a decrease was seen after 5 h (Molenat et al., 2004; Boussuges et al., 2007). In the present study, we observed a rapid and significant decrease in CO after 2.5 min at 600 kPa. Since heart rate remained unchanged, the reduction of CO in our study was explained by a decrease in SV, in accordance with the earlier findings (Molenat et al., 2004). Our observations are supported by Stuhr et al. (1989) who observed pronounced changes in cardiac function at an ambient pressure of 500 kPa. The reduced SV took place in parallel with the increase in LV end-systolic volume, whereas LV end-diastolic volume remained unchanged. This in support of our interpretation that the reduced SV was a consequence of the abrupt increase in afterload. Further, the unchanged LV end-diastolic volume indicated that pulmonary artery pressure did not increase (Valic et al., 2005) to a level which compromised LV filling. This may imply that right ventricular function and LV preload remained unaltered at pressure in our experiment.

Due to a 70% increase in vascular resistance during the bottom phase, MAP, and thus cardiac afterload, increased significantly. The product of HR and MAP, also termed the double product, is often used to determine stress put on the cardiac muscle during exercise as it correlates with changes in myocardial oxygen consumption (MVO_2_) (Amsterdam and Mason, 1977). Due to physiologic baroreflex stabilization during exercise; if afterload is increased abruptly, HR will fall, and vice versa (van Vliet and Montani, 1999). In general, the double product remains relatively unchanged during exercise, interpreted as a way to save MVO_2_ during cardiac stress. In the present experiment, however, the 30% increase in MAP is not compensated by a fall in HR. This may represent an abrupt increase in MVO_2_, which is well tolerated by the healthy heart, but could be a risk factor in the presence of coronary artery disease or myocardial failure. The partial pressure of oxygen at the bottom phase in our study was 120 kPa, and the increased MAP could be caused by hyperoxia-induced vasoconstriction (Mak et al., 2001; Waring et al., 2003) leading to an increased in vascular resistance (Milone et al., 1999; Waring et al., 2003). This is in line with increased MAP accompanied with increased TPR and LV end systolic pressure in the present study, indicating increased LV afterload.

By monitoring LV dP/dt_max_, some have reported increased myocardial contractility with increase in ambient pressure (Stuhr et al., 1989; Risberg et al., 1995). However, LV dP/dt_max_ is sensitive to changes in pre- and after-load, which can vary considerably during compression/decompression. This was observed in response to diving in our experiment, making this variable a less reliable index of LV contractility under the present circumstances. But, based on continuous measurements of LV dP/dt_max_ and on PRSW calculated before and after the simulated diving exposure, we are able to conclude that in our experiment all changes in LV function were reversible by decompression.

The protocol for the current study required the animals to be anesthetized, and anesthesia affects vascular tone (Matsukawa et al., 1995). In pilot studies at our laboratory, anesthetized rats did not survive decompression from our usual air dive protocol to 700 kPa. Thus, since we wanted to observe the rats for 60 min after decompression, bottom pressure was reduced to 600 kPa. Future studies are needed to determine why anesthetized rats are more vulnerable to decompression stress.

Our diving rats experienced a drop in respiratory rate during the bottom phase. At high ambient pressure, respiratory function is affected by increased resistive and elastic load, and increased partial pressures of inert gas and oxygen (Moon et al., 2009). As we did not perform respiratory functional measurements we have no additional data to explain the observed decrease in respiratory rate, but other studies have reported decreased alveolar-arterial partial pressure of oxygen difference at high gas densities (Christopherson and Hlastala, 1982; Moon et al., 2009), possibly caused by altered blood flow distribution resulting in a more efficient ventilation perfusion ratio.

Our experiments were performed in a dry pressure chamber, which limits the general interpretation of the result. Dry dives are not directly comparable to SCUBA diving where water immersion induces prolonged cardiovascular changes (Marabotti et al., 1999, 2013; Boussuges et al., 2009), and influences bubble production and DCS risk (Obad et al., 2007; Gaustad et al., 2010). To improve the understanding of cardiovascular responses to wet diving, future studies should include LV pressure–volume recording in immersed animals. It should also be noted that only female rats were included our study. While cardiovascular anatomy is similar for females and males, there are sex differences in cardiovascular function (Huxley, 2007), and we cannot speculate whether the responses would be identical in males.

## Conclusion

In conclusions, our simulated dive experiment to 600 kPa increased MAP and TRP to levels which caused a substantial reduction in SV and LV volume output. The elevated cardiac stress which takes place during a dive, here demonstrated by the increase in afterload, is well tolerated by the healthy heart but may lead to acute cardiac decompensation in a failing heart.

## Data Availability Statement

The datasets generated for this study are available on request to the corresponding author.

## Ethics Statement

The animal study was reviewed and approved by the Norwegian Animal Research Authority.

## Author Contributions

SG designed the study, and contributed to the experimental work and manuscript writing. TK contributed to the experimental work and statistical analysis. IE contributed to the manuscript writing. TT contributed to the study design and manuscript writing. All authors contributed and approved the final version of the manuscript.

## Conflict of Interest

SG was employed by the company Møreforskning AS. The remaining authors declare that the research was conducted in the absence of any commercial or financial relationships that could be construed as a potential conflict of interest.
